# Life satisfaction among older adults in China: who gains more from community-based health services?

**DOI:** 10.3389/fpubh.2025.1705076

**Published:** 2025-12-05

**Authors:** Cangcang Jia, Ling Zhang, Zhengyang Wang, Zhiguang Li

**Affiliations:** 1 Laboratory for Digital Intelligence and Health Governance, Nanjing Medical University, Nanjing, China; 2 School of Health Policy and Management, Nanjing Medical University, Nanjing, China; 3 Center for Health Policy and Management Studies, School of Government, Nanjing University, Nanjing, China; 4 The First Affiliated Hospital, Nanjing Medical University, Nanjing, China

**Keywords:** life satisfaction, community-based health services, older adults, health policy, China

## Abstract

**Objectives:**

This study investigates the effects of community-based health services on life satisfaction among older adults in China, examining three specific service types: health check-ups, health records, and health education programs.

**Methods:**

A cross-sectional quantitative study utilizing data from the China Longitudinal Aging Social Survey (CLASS) conducted in 2020. Ordinary Least Squares (OLS) regression models were employed for the benchmark analysis, with ordered logit and ordered probit models used for robustness verification. Propensity Score Matching (PSM) method with multiple algorithms was adopted to address potential selection bias and strengthen the basis for causal inference. Variable substitution and sample replacement methods were implemented for additional robustness tests. Heterogeneity analyses were conducted to examine differential effects across demographic subgroups.

**Results:**

The analysis included 11,292 older adults aged 60 and above. Health check-up services showed the highest utilization rate (29.93%), followed by health records (10.85%) and health education (7.03%). All three community-based health services showed significant positive associations with life satisfaction, with relationships that remained statistically significant after rigorous statistical adjustment for observable confounders. The positive effects remained consistent across different model specifications and robustness checks. Heterogeneity analyses revealed that female, rural, and chronically ill older adults experienced greater benefits from these services compared to their counterparts.

**Conclusion:**

Community-based health services show strong positive associations with enhanced life satisfaction among older adults in China, with differential impacts across population subgroups. These findings provide evidence for expanding community-based health service coverage and developing targeted interventions for vulnerable older populations, particularly focusing on rural areas and individuals with chronic conditions to maximize program effectiveness.

## 1 Introduction

The global phenomenon of population aging presents formidable challenges to healthcare systems worldwide, straining their capacity to deliver accessible and affordable care services for the rapidly growing older population (1, 2). China faces an unprecedented demographic challenge as the population aged 60 and above is approximately 310 million individuals, accounting for about 21.9% of China’s population in 2024 (3, 4). This demographic transformation occurs at a pace that far exceeds that experienced by developed nations, with China’s aging rate projected to reach 33% by 2050, compared to the gradual transitions witnessed in Europe and North America over several decades. The socioeconomic implications of this shift are profound, particularly as approximately 75% of Chinese older adults suffer from two or more chronic diseases (5–7). This rapid aging trend necessitates urgent development of innovative care models to preserve health outcomes and quality of life for this expanding population segment.

In this rapidly aging landscape, traditional family-based care systems are increasingly unable to meet older adults’ comprehensive wellbeing needs, contributing to declining life satisfaction among this vulnerable population (8). The legacy of China’s one-child policy has resulted in a “4–2-1” family structure, where a single adult child is often responsible for caring for multiple older family members while managing their own responsibilities, leading to inadequate attention and emotional support for older adults (9–11). This care deficit directly impacts the wellbeing of older adults, as insufficient family support has been associated with increased social isolation, reduced sense of security, and lower overall life satisfaction among older adults. The economic burden of managing chronic conditions not only strains family resources but also creates stress and anxiety for older adults who worry about being financial burdens on their children, further diminishing their subjective wellbeing (12–14). Moreover, the lack of professional support and specialized care knowledge within families often results in suboptimal health management and unmet psychosocial needs, contributing to decreased quality of life and life satisfaction among older adults (15).

To address these mounting challenges, the Chinese government has initiated a major policy transformation toward community-based health services. In 2009, China incorporated health services for all individuals aged 65 and above into the National Basic Public Health Service Program, with continuous optimization and supplementation in subsequent years (16, 17). This community-level implementation encompasses three core components to support aging in place: preventive health examinations, systematic health records management, and targeted health education program. The policy aims to improve health outcomes and subjective wellbeing among older adults while alleviating pressure on families and the formal healthcare system (18). Despite the policy priority placed on these services, current utilization patterns reveal significant implementation challenges and untapped potential. Based on our analysis of 11,292 older adults from the 2020 China Longitudinal Aging Social Survey, health check-up services demonstrate the highest utilization rate at 29.93% (3,380 respondents), followed by health records establishment at 10.85% (1,225 respondents), and health education programs at only 7.03% (794 respondents). These utilization disparities are particularly concerning given that health education represents one of the most cost-effective preventive interventions for healthy aging. The low uptake of health education services, despite their potential for broad population impact, highlights critical gaps in service delivery mechanisms and accessibility that warrant urgent policy attention. These utilization patterns underscore the need for comprehensive evaluation of service effectiveness and the development of targeted strategies to optimize community health service delivery for China’s rapidly aging population.

Life satisfaction serves as a critical outcome measure as it reflects an older adult’s holistic evaluation of their quality of life across multiple domains, including health, social relationships, and economic security (19, 20). For Chinese older adults, life satisfaction is determined by a complex interplay of factors extending beyond economic resources to encompass health status, social support networks, and the quality of the community environment, where their daily activities are increasingly concentrated (21–23). Strong community cohesion and robust support networks are particularly significant enhancers of wellbeing. However, this is substantially challenged by the high prevalence of multimorbidity, which affects a large segment of the older population and poses a significant threat to their subjective wellbeing (24, 25). Furthermore, factors such as gender, age, and urban–rural residence create differential patterns in life satisfaction that warrant careful investigation.

Despite a growing international literature on community-based health interventions, significant research gaps persist regarding their impact on subjective wellbeing in the Chinese context. Much of the existing research in China is descriptive, lacking the rigorous causal inference methods needed to establish clear effects. Furthermore, the heterogeneous impacts of these services—particularly across the rural–urban divide, between genders, and among individuals with different health statuses—remain largely unexplored (26). This study addresses these critical gaps by using data from the China Longitudinal Aging Social Survey (CLASS) to conduct a comprehensive, multi-dimensional analysis of how community-based health services influence life satisfaction among older adults. Community-based health services align closely with established theoretical frameworks of healthy and active aging. The World Health Organization’s Healthy Aging framework emphasizes optimizing opportunities for health, participation, and security to enhance quality of life as people age (27). Similarly, the Active Aging paradigm promotes health, participation, and security as interconnected determinants of successful aging (28). These frameworks provide the theoretical foundation for understanding how community-based health interventions can support older adults’ wellbeing through multiple pathways, including health maintenance, social engagement, and enhanced autonomy.

This study makes two primary innovative contributions that advance understanding of community-based health services and life satisfaction among older adults. First, while most existing studies examine community health services as a single intervention, our research systematically disaggregates these services into three specific components (health check-ups, health records, and health education) and evaluates their differential impacts on life satisfaction. Second, we conduct comprehensive heterogeneity analyses across multiple demographic dimensions simultaneously (gender, urban–rural residence, chronic disease status, and age groups), revealing nuanced patterns of service effectiveness that have been largely overlooked in prior research. The synergy between these two innovations enables identification of not only which specific services are most effective, but also for whom they work best, facilitating precise policy targeting and resource allocation. These primary innovations are supported by rigorous causal inference methodology through propensity score matching with multiple algorithms, addressing selection bias limitations that have compromised previous studies, and the use of nationally representative CLASS data that provides generalizable insights for China’s entire older population.

## 2 Methods

### 2.1 Data and sampling

This study utilized micro-level data from the China Longitudinal Aging Social Survey (CLASS) to examine the impact of community-based health services on life satisfaction among older adults. The CLASS is a nationally representative survey jointly designed and implemented by the Institute of Gerontology at Renmin University of China. The survey employs a stratified multi-stage probability sampling design, covering over 30 provinces, more than 400 village-level units, and over 11,000 individuals aged 60 and above, ensuring excellent national representativeness of the data. The CLASS questionnaire encompasses a wealth of information regarding the socioeconomic status of older adults, as well as health services, social security, and community support, providing sufficient samples and appropriate indicators for the research on aging issues in China. The latest data from the 2020 wave has been selected as the empirical analysis samples for this article. Our analytical sample comprises 11,292 older adult respondents, among whom 3,380 (29.9%) received free health examinations, 1,225 (10.9%) had health records established, and 794 (7.0%) participated in health education programs. This variation in community health service utilization provides an ideal setting for identifying their impacts on life satisfaction among older adults.

### 2.2 Variables definition

#### 2.2.1 The dependent variable

The dependent variable in this study is the life satisfaction of older adults, measured by the item B17 in the CLASS 2020 questionnaire: “Overall, are you satisfied with your current life?.” In the original survey, responses were coded on a 5-point scale where 1 indicated “very satisfied,” and 5 indicated “very dissatisfied.” For ease of interpretation in the analysis, this scale was reverse-coded. Therefore, in this study, the variable is treated as an ordered measure where a higher value represents a greater level of life satisfaction (1 = very dissatisfied, 2 = relatively dissatisfied, 3 = average, 4 = relatively satisfied, and 5 = very satisfied).

#### 2.2.2 Independent variable

Community-based health services are defined as a comprehensive set of preventive healthcare interventions delivered at the community level to support healthy aging among older adults. Drawing on the theoretical framework of community-based healthcare delivery systems and existing empirical evidence, this study operationalizes community health services through three distinct yet complementary dimensions that collectively capture the multifaceted nature of community-level health promotion for older populations.

The first dimension is free health examination services, which encompasses systematic health assessments and preventive screening programs provided at the community level. Health examinations serve as a fundamental component of preventive healthcare, enabling early detection of health conditions, monitoring of chronic disease progression, and establishment of baseline health profiles for ongoing care management (29). This dimension captures whether respondents utilized community-provided free health examination services in the past 12 months, coded as a dummy variable where 1 indicates utilization and 0 indicates non-utilization.

The second dimension is health record establishment services, which represents the systematic documentation and management of individual health information within community-based care systems. Health records serve as the foundational infrastructure for continualized care delivery, enabling community healthcare providers to function as “health managers” for community-dwelling older adults (8). By maintaining comprehensive health profiles that include medical history, current health status, medication usage, and care preferences, health records facilitate coordinated care planning, enable tracking of health trajectories over time, and support evidence-based decision-making in community health interventions. This service dimension reflects the transition from episodic to continuous care models, which is particularly crucial for managing the complex health needs of aging populations. This dimension captures whether respondents utilized community-provided health record establishment services in the past 12 months, similarly coded as a dummy variable.

The third dimension is health education services, which constitutes a cornerstone of public health interventions designed to provide sustained coordinated care through health education, lifestyle modification, and behavioral support. Community-based health education programs have become essential components of preventive healthcare delivery, particularly in resource-limited primary healthcare settings where such non-pharmacological interventions provide sustainable, long-term health management support for high-risk populations (30). Existing empirical evidence demonstrates that community-based health education programs can effectively help individuals foster healthier habits, control chronic disease risk factors such as blood pressure and lipid profiles, and improve health behaviors including smoking cessation rates (31). These educational interventions address not only clinical health indicators but also health literacy, self-care capabilities, and health behavior modification, thereby addressing the broader determinants of health and wellbeing among older populations. This dimension captures whether respondents utilized community-provided health education services in the past 12 months, coded using the same dummy variable approach.

These three dimensions collectively represent different yet complementary aspects of preventive healthcare delivery at the community level, each with distinct theoretical contributions to the wellbeing of older adults. Specifically, (a) health check-ups function as preventive clinical assessment and early detection interventions, providing immediate health status evaluation and establishing baseline health profiles for ongoing care management; (b) health records facilitate care continuity and coordination by enabling longitudinal health monitoring and systematic tracking of health conditions over time, representing the crucial transition from episodic to continuous care models; and (c) health education addresses behavioral and lifestyle factors through health literacy enhancement, self-care capability building, and health behavior modification, serving as cornerstone interventions for sustainable long-term health management that extend beyond clinical indicators. Together, these three service dimensions provide a comprehensive framework for community-based health service utilization that aligns with multifaceted approaches to healthy aging promotion (32).

#### 2.2.3 Control variables

To mitigate the endogeneity problem caused by omitted variables, various covariates that affect the life satisfaction of older adults are included in this study, including demographic characteristics (age, gender, marriage), socioeconomic characteristics (education, residence, living arrangement, economic position, work status), and health-related characteristics (chronic disease, self-rated health, exercise, body mass index), all of which were collected through the CLASS structured questionnaire. Age is a continuous variable measured in years. According to the World Health Organization and previous studies (29, 33, 40), participants were categorized into two age ranges: 60 to 79 (*N* = 9,692), 80 and above 80 (*N* = 1,600). Gender is a dummy variable, with 1 representing male and 0 representing female. Marriage is a dummy variable, with 1 indicating currently married with spouse present and 0 indicating other status including widowed, divorced, and never married. Educational attainment is captured using dummy variables for primary school, middle school, and high school or above, with illiteracy serving as the reference category. Place of residence is a dummy variable, with 1 indicating urban areas and 0 indicating rural areas. Living arrangement is a dummy variable, with 1 indicating non-living alone and 0 indicating living alone. Economic position captures relative economic status through the question “Compared with people around you, how do you think your economic situation is?” with responses coded as 0 = worse, 1 = similar, 2 = better. Work status indicates current engagement in income-generating activities, including agricultural labor where agricultural products can be converted to income, coded as 0 = not participating, 1 = participating. Chronic disease is a dummy variable, with 1 indicating the presence of any diagnosed chronic condition and 0 indicating absence. Self-rated health is measured on a five-point Likert scale, ranging from 1 (very unhealthy) to 5 (very healthy). Physical exercise is a dummy variable, with 1 indicating regular participation in exercise and 0 indicating no regular participation. Body mass index (BMI) is a continuous variable calculated from self-reported height and weight according to the Chinese national health industry standard WS/T428-2013 “Guidelines for the Prevention and Control of Overweight and Obesity in Adults.” We categorized BMI as underweight (BMI < 18.5), normal weight (18.5 ≤ BMI < 24.0), overweight (24.0 ≤ BMI < 28.0), and obese (BMI ≥ 28.0) for descriptive analysis. Descriptive statistics for these variables are presented in Table 1.

**Table 1:** Characteristics of the study samples, CLASS 2020.

Variables	Mean	SD	Min	Max
Life satisfaction (1 = very dissatisfied; 2 = relatively dissatisfied; 3 = average; 4 = relatively satisfied; 5 = very satisfied)	3.7236	0.8916	1	5
Community-based health services				
Health check-up (0 = no, 1 = yes)	0.2993	0.4579	0	1
Health records (0 = no, 1 = yes)	0.1085	0.3110	0	1
Health education (0 = no, 1 = yes)	0.0703	0.2556	0	1
Age (years old)	71.5708	6.5898	60	98
60–79 age group (0 = no, 1 = yes)	0.8583	0.3487	0	1
80 and above 80 age group (0 = no, 1 = yes)	0.1417	0.3487	0	1
Gender (0 = female; 1 = male)	0.5046	0.5000	0	1
Marriage (0 = unmarried; 1 = married)	0.7547	0.4302	0	1
Education				
Illiteracy (0 = no, 1 = yes)	0.2742	0.4461	0	1
Primary school (0 = no, 1 = yes)	0.3689	0.4825	0	1
Middle school (0 = no, 1 = yes)	0.2478	0.4317	0	1
High school and above	0.1091	0.3117	0	1
Residential region (0 = rural; 1 = urban)	0.5082	0.4999	0	1
Economic status (0 = worse; 1 = similar; 2 = better)	1.0658	0.5405	0	2
Work (0 = no; 1 = yes)	0.2597	0.4385	0	1
Living arrangement (0 = living alone; 1 = non-living alone)	0.8981	0.3024	0	1
Chronic disease (0 = no, 1 = yes)	0.2139	0.4101	0	1
Self-rated health (1 = very bad; 2 = bad; 3 = so so; 4 = good; 5 = very good)	3.3716	0.8981	1	5
Exercise (0 = no, 1 = yes)	0.4009	0.4901	0	1
Body mass index (BMI)				
Underweight (0 = no, 1 = yes)	0.0516	0.2212	0	1
Normal weight (0 = no, 1 = yes)	0.6390	0.4803	0	1
Overweight (0 = no, 1 = yes)	0.2740	0.4460	0	1
Obese (0 = no, 1 = yes)	0.0354	0.1846	0	1

### 2.3 Statistical analyses

In this study, the OLS model estimation was used to construct a baseline regression model for the purpose of examining the relationship between the community-based health services (CBHS) and older adults’ life satisfaction. The following is a description of the model:


$$\text{Sati}s_{i}=\alpha +\beta_{1}\mathrm{CBH}S_{i}+\beta_{2}X_{i}^{’}+\varepsilon_{i}$$ (1)

In Equation 1, $\text{Sati}s_{i}$ is the dependent variable reflecting the life satisfaction of older adults. The independent variable $\mathrm{CBH}S_{i}$ indicates whether older adults use community-based health services. $X_{i}^{’}$ representative the relevant control variables, and β_1_ is the coefficient to be estimated, and its numerical value reflects the relationship between the community-based health services and life satisfaction among older adults based on whether or not it is significant. $\varepsilon_{i}$ a random perturbation term.

The potential for selection bias in community health service utilization arises through several systematic pathways that may confound the relationship between service use and life satisfaction outcomes. Older adults with higher health consciousness and proactive health-seeking behaviors are more likely to utilize preventive community health services, and these individuals may also engage in other health-promoting activities and lifestyle choices that independently contribute to higher life satisfaction, creating potential upward bias in estimated effects. Similarly, despite public funding, community health services may be more accessible to older adults with better economic resources, transportation access, or flexible schedules, and higher socioeconomic status independently predicts greater life satisfaction through multiple pathways including reduced financial stress, better living conditions, and enhanced social participation. Geographic factors also contribute to selection bias, as older adults residing in areas with well-developed community health infrastructure are more likely to receive services, while these same communities often have superior overall social services, recreational facilities, and social cohesion that may independently enhance residents’ life satisfaction. Additional selection mechanisms include social capital and baseline wellbeing effects, where older adults with stronger social networks and higher education levels are more likely to be aware of and successfully navigate community health services, while these social resources also directly contribute to life satisfaction through enhanced social support and community engagement. Furthermore, older adults with higher baseline life satisfaction may be more motivated to engage with community services as part of maintaining their wellbeing, while those with depression or social isolation may be less likely to participate despite potentially benefiting most from these interventions. This study employs the propensity score matching (PSM) method to mitigate the potential selectivity bias of CBHS on the older population’s life satisfaction. The propensity score for each older adult is computed based on the identified confounding variables using logistic regression. Multiple matching algorithms were employed, including k-nearest neighbor matching (*k* = 4), radius matching (caliper = 0.01), kernel matching, and Mahalanobis matching to ensure robustness of results. The average treatment effect on the treated (ATT) was calculated for each matching method.

For additional robustness checks, several strategies were implemented: (1) alternative model specifications including ordered probit and ordered logit models; (2) variable substitution tests; (3) sample replacement analysis. Following (39), the sign and significance differences between ordered models and OLS are minimal, supporting the use of OLS for intuitive interpretation.

Balance tests were conducted using pstest to evaluate covariate balance before and after matching, with standardized differences less than 5% considered as good balance. Heterogeneity analysis was performed across different subgroups including gender, chronic disease status, age groups, and other demographic characteristics.

All statistical analyses were performed using Stata SE 18.0 software. Statistical significance was set at *p* < 0.05, with 95% confidence intervals reported for all estimates.

## 3 Results

### 3.1 The characteristics of the study samples

The characteristics of the 11,292 participants from the CLASS 2020 survey reveal important patterns in community health service utilization that have significant policy implications. The average age of respondents was 71.57 years (SD = 6.59), with 50.5% being male. Among the three community-based health services examined, pronounced utilization disparities emerged. Health check-up services achieved the highest uptake at 29.93% (*n* = 3,380), followed by health records establishment at 10.85% (*n* = 1,225), and health education programs at only 7.03% (*n* = 794). This utilization gradient is particularly striking given that health education programs, despite having the lowest participation rates, demonstrated substantial effectiveness in our subsequent analyses. The limited reach of health education services represents a significant missed opportunity for preventive health promotion among older adults, highlighting critical gaps in current service delivery approaches that require targeted policy interventions to enhance accessibility and uptake. Participants reported a mean life satisfaction score of 3.72 (SD = 0.89) and an average self-rated health score of 3.37 (SD = 0.90), with 21.4% reporting at least one chronic disease. The sample was predominantly married (75.5%), lived with others (89.8%), and was nearly balanced between urban and rural areas, with primary school being the most common educational attainment.

### 3.2 Effects of community-based health services on the life satisfaction of older adults

The regression results demonstrate that all three types of community-based health services consistently exhibit significant positive effects on older adults’ life satisfaction across different model specifications. The estimated coefficients of health check-up services, health records services, and health education services all demonstrate statistically significant positive values in the OLS models. The ordered logit and ordered probit models yield consistent results with similar patterns of significance, confirming the robustness of these findings. These results provide strong empirical evidence that community-based health services significantly enhance the life satisfaction of older adults, with health education and health records showing slightly larger effects compared to health check-up services, though all three services demonstrate substantial positive impact (Table 2).

**Table 2:** OLS, Ologit, and Oprobit results of community-based health services on the life satisfaction among older adults.

Variables	Life satisfaction among older adults								
	OLS model			Ologit model			Oprobit model		
Health check-up	0.1067^***^			0.2592^***^			0.1579^***^		
	[0.0737, 0.1398]			[0.1800, 0.3384]			[0.1128, 0.2031]		
Health records		0.1481^***^			0.3632^***^			0.2204^***^	
		[0.0942, 0.2020]			[0.2310, 0.4955]			[0.1467, 0.2941]	
Health education			0.1490^***^			0.3117^***^			0.1980^***^
			[0.0901, 0.2078]			[0.1718, 0.4515]			[0.1177, 0.2784]
80 and above 80 age group	0.0144	0.0190	0.0215	0.0582	0.0685	0.0734	0.0291	0.0359	0.0389
	[−0.0329, 0.0618]	[−0.0282, 0.0663]	[−0.0258, 0.0689]	[−0.0524, 0.1687]	[−0.0417, 0.1787]	[−0.0371, 0.1839]	[−0.0336, 0.0917]	[−0.0266, 0.0985]	[−0.0238, 0.1016]
Gender	−0.0252	−0.0269^*^	−0.0262^*^	−0.0627^*^	−0.0675^*^	−0.0661^*^	−0.0353^*^	−0.0378^*^	−0.0367^*^
	[−0.0556, 0.0053]	[−0.0573, 0.0036]	[−0.0567, 0.0042]	[−0.1349, 0.0095]	[−0.1397, 0.0047]	[−0.1382, 0.0061]	[−0.0768, 0.0062]	[−0.0793, 0.0037]	[−0.0782, 0.0048]
Marriage	0.0531^**^	0.0508^**^	0.0516^**^	0.1232^**^	0.1157^**^	0.1189^**^	0.0778^***^	0.0744^**^	0.0751^**^
	[0.0089, 0.0974]	[0.0065, 0.0951]	[0.0072, 0.0959]	[0.0204, 0.2259]	[0.0129, 0.2186]	[0.0161, 0.2216]	[0.0194, 0.1362]	[0.0160, 0.1328]	[0.0167, 0.1336]
Primary school	−0.0385^*^	−0.0401^**^	−0.0381^*^	−0.0826^*^	−0.0876^*^	−0.0820^*^	−0.0522^*^	−0.0548^**^	−0.0512^*^
	[−0.0777, 0.0008]	[−0.0793, −0.0008]	[−0.0774, 0.0012]	[−0.1756, 0.0104]	[−0.1807, 0.0055]	[−0.1749, 0.0110]	[−0.1049, 0.0006]	[−0.1076, −0.0019]	[−0.1040, 0.0016]
Middle school	−0.0106	−0.0145	−0.0108	−0.0185	−0.0293	−0.0200	−0.0162	−0.0222	−0.0163
	[−0.0557, 0.0345]	[−0.0596, 0.0306]	[−0.0560, 0.0343]	[−0.1250, 0.0879]	[−0.1359, 0.0772]	[−0.1264, 0.0865]	[−0.0774, 0.0449]	[−0.0833, 0.0389]	[−0.0774, 0.0448]
High school and above	0.0878^***^	0.0827^***^	0.0917^***^	0.2517^***^	0.2376^***^	0.2593^***^	0.1372^***^	0.1302^***^	0.1437^***^
	[0.0317, 0.1438]	[0.0267, 0.1388]	[0.0357, 0.1478]	[0.1142, 0.3892]	[0.1002, 0.3749]	[0.1221, 0.3965]	[0.0585, 0.2159]	[0.0515, 0.2089]	[0.0651, 0.2222]
Residential region	0.0664^***^	0.0655^***^	0.0558^***^	0.1889^***^	0.1885^***^	0.1645^***^	0.0899^***^	0.0888^***^	0.0750^***^
	[0.0323, 0.1004]	[0.0314, 0.0995]	[0.0219, 0.0898]	[0.1077, 0.2700]	[0.1071, 0.2700]	[0.0834, 0.2455]	[0.0436, 0.1362]	[0.0423, 0.1352]	[0.0288, 0.1213]
Economic status work	0.0755^***^	0.0724^***^	0.0728^***^	0.1586^***^	0.1498^***^	0.1513^***^	0.0980^***^	0.0933^***^	0.0937^***^
	[0.0461, 0.1050]	[0.0429, 0.1018]	[0.0433, 0.1023]	[0.0883, 0.2289]	[0.0796, 0.2200]	[0.0810, 0.2216]	[0.0582, 0.1378]	[0.0535, 0.1330]	[0.0538, 0.1336]
	0.0317^*^	0.0337^*^	0.0299	0.0482	0.0560	0.0444	0.0361	0.0394	0.0328
	[−0.0040, 0.0674]	[−0.0022, 0.0695]	[−0.0059, 0.0657]	[−0.0378, 0.1341]	[−0.0307, 0.1426]	[−0.0419, 0.1306]	[−0.0132, 0.0854]	[−0.0102, 0.0889]	[−0.0166, 0.0822]
Living arrangement	0.0830^***^	0.0819^***^	0.0831^***^	0.2092^***^	0.2066^***^	0.2086^***^	0.1097^***^	0.1080^***^	0.1103^***^
	[0.0223, 0.1437]	[0.0211, 0.1427]	[0.0222, 0.1441]	[0.0690, 0.3493]	[0.0663, 0.3470]	[0.0680, 0.3493]	[0.0294, 0.1900]	[0.0277, 0.1884]	[0.0299, 0.1908]
Chronic disease	0.1134^***^	0.1062^***^	0.1054^***^	0.2908^***^	0.2698^***^	0.2674^***^	0.1722^***^	0.1616^***^	0.1594^***^
	[0.0768, 0.1499]	[0.0700, 0.1424]	[0.0691, 0.1417]	[0.1995, 0.3820]	[0.1797, 0.3600]	[0.1771, 0.3577]	[0.1197, 0.2247]	[0.1095, 0.2136]	[0.1073, 0.2115]
Self-rated health	0.4056^***^	0.4102^***^	0.4090^***^	1.0093^***^	1.0214^***^	1.0184^***^	0.5342^***^	0.5406^***^	0.5380^***^
	[0.3847, 0.4265]	[0.3894, 0.4309]	[0.3882, 0.4299]	[0.9514, 1.0673]	[0.9637, 1.0791]	[0.9605, 1.0762]	[0.5036, 0.5647]	[0.5102, 0.5710]	[0.5075, 0.5686]
Exercise	−0.0087	−0.0117	−0.0140	−0.0243	−0.0349	−0.0366	−0.0150	−0.0187	−0.0216
	[−0.0402, 0.0227]	[−0.0432, 0.0199]	[−0.0456, 0.0176]	[−0.0989, 0.0504]	[−0.1099, 0.0401]	[−0.1115, 0.0384]	[−0.0578, 0.0278]	[−0.0617, 0.0242]	[−0.0646, 0.0214]
Underweight	−0.0932^**^	−0.0901^**^	−0.0869^**^	−0.1993^**^	−0.1905^**^	−0.1832^**^	−0.1017^*^	−0.0979^*^	−0.0937^*^
	[−0.1710, −0.0155]	[−0.1684, −0.0117]	[−0.1652, −0.0085]	[−0.3793, 0.0193]	[−0.3725, −0.0084]	[−0.3648, −0.0016]	[−0.2039, 0.0006]	[−0.2009, 0.0051]	[−0.1967, 0.0093]
Overweight	0.0790^***^	0.0786^***^	0.0767^***^	0.1951^***^	0.1914^***^	0.1891^***^	0.1142^***^	0.1142^***^	0.1109^***^
	[0.0453, 0.1127]	[0.0450, 0.1122]	[0.0431, 0.1104]	[0.1136, 0.2766]	[0.1102, 0.2725]	[0.1077, 0.2704]	[0.0677, 0.1607]	[0.0678, 0.1605]	[0.0644, 0.1573]
Obese	−0.0077	−0.0050	−0.0121	0.0183	0.0183	0.0056	0.0090	0.0140	0.0031
	[−0.0932, 0.0779]	[−0.0898, 0.0798]	[−0.0973, 0.0732]	[−0.1871, 0.2237]	[−0.1850, 0.2216]	[−0.1989, 0.2100]	[−0.1044, 0.1224]	[−0.0985, 0.1265]	[−0.1099, 0.1161]
_cons	2.0677^***^	2.0790^***^	2.0906^***^						
	[1.9696, 2.1658]	[1.9812, 2.1768]	[1.9923, 2.1889]						
Cut1				−0.3811^***^	−0.4119^***^	−0.4404^***^	−0.2496^***^	−0.2667^***^	−0.2897^***^
				[−0.6495,-0.1128]	[−0.6797,-0.1441]	[−0.7090,-0.1718]	[−0.3861,-0.1131]	[−0.4026,-0.1308]	[−0.4260,-0.1533]
Cut2				1.4561^***^	1.4269^***^	1.3992^***^	0.6592^***^	0.6425^***^	0.6215^***^
				[1.2162, 1.6960]	[1.1872, 1.6665]	[1.1588, 1.6395]	[0.5277, 0.7908]	[0.5116, 0.7735]	[0.4901, 0.7530]
Cut3				3.4144^***^	3.3849^***^	3.3578^***^	1.7677^***^	1.7506^***^	1.7300^***^
				[3.1663, 3.6624]	[3.1372, 3.6326]	[3.1093, 3.6062]	[1.6310, 1.9044]	[1.6146, 1.8867]	[1.5935, 1.8666]
Cut4				5.9595^***^	5.9283^***^	5.8973^***^	3.2490^***^	3.2313^***^	3.2081^***^
				[5.6843, 6.2347]	[5.6536, 6.2030]	[5.6217, 6.1729]	[3.1012, 3.3968]	[3.0843, 3.3783]	[3.0604, 3.3557]
*N*	11,292	11,292	11,292	11,292	11,292	11,292	11,292	11,292	11,292
*R* ^2^	0.1986	0.1983	0.1975						
Adj. *R*^2^	0.1974	0.1971	0.1962						

95% confidence intervals in brackets. ^*^p < 0.1, ^**^p < 0.05, ^***^p < 0.01.

### 3.3 Robustness test

#### 3.3.1 Robustness test for substituting variables

To ensure the reliability of our findings and address potential concerns about variable measurement sensitivity, we conducted a robustness check by systematically altering the operationalization of our dependent variable. Our benchmark analysis utilized a five-point ordinal scale measuring life satisfaction intensity, with responses ranging from very dissatisfied to very satisfied. To validate that our results are not dependent on this specific measurement approach, we transformed the dependent variable into a dichotomous measure that captures satisfaction status rather than satisfaction intensity. This binary transformation categorizes older adults into two distinct groups based on their life satisfaction evaluations. The “satisfied” group (coded as 1) encompasses respondents expressing positive life satisfaction, specifically those reporting being “relatively satisfied” or “very satisfied” with their current life circumstances. Conversely, the “not satisfied” group (coded as 0) includes all remaining respondents, comprising those who reported “average,” “relatively dissatisfied,” or “very dissatisfied” responses. This coding scheme adopts a conservative threshold where neutral evaluations are classified alongside negative responses, ensuring that only genuinely positive satisfaction assessments are captured in the satisfied category. As shown in Table 3, the results from this alternative specification confirm that each type of community-based health service continues to demonstrate statistically significant positive associations with older adults’ satisfaction outcomes. This consistency across different measurement approaches strengthens confidence in the robustness of our primary findings.

**Table 3:** Robustness test results using restricted sample of older adults with expressed interest in community-based health services.

Variables	Life satisfaction among older adults					
	Substituting variables			Replacing sample		
Health check-up	0.0588^***^			0.1536^***^		
	[0.0412, 0.0764]			[0.1098, 0.1974]		
Health records		0.0510^***^			0.2291^***^	
		[0.0243, 0.0778]			[0.1621, 0.2961]	
Health education			0.0746^***^			0.2253^***^
			[0.0431, 0.1062]			[0.1510, 0.2996]
80 and above 80 age group	−0.0014	0.0012	0.0024	0.0408	0.1355^***^	0.0462
	[−0.0267, 0.0238]	[−0.0240, 0.0264]	[−0.0229, 0.0276]	[−0.0239, 0.1054]	[0.0336, 0.2375]	[−0.0682, 0.1607]
Gender	−0.0067	−0.0077	−0.0073	−0.0387^*^	−0.0359	−0.0038
	[−0.0233, 0.0100]	[−0.0244, 0.0090]	[−0.0240, 0.0094]	[−0.0828, 0.0054]	[−0.0996, 0.0278]	[−0.0741, 0.0666]
Marriage	0.0128	0.0113	0.0118	0.0481	0.0211	0.0855
	[−0.0112, 0.0367]	[−0.0127, 0.0353]	[−0.0122, 0.0358]	[−0.0138, 0.1099]	[−0.0694, 0.1116]	[−0.0170, 0.1881]
Primary school	−0.0234^**^	−0.0231^**^	−0.0230^**^	0.0017	0.0033	0.0479
	[−0.0451, −0.0017]	[−0.0448, −0.0014]	[−0.0447, −0.0013]	[−0.0551, 0.0585]	[−0.0880, 0.0947]	[−0.0554, 0.1513]
Middle school	0.0129	0.0122	0.0130	0.0460	0.1057^**^	0.1339^**^
	[−0.0116, 0.0375]	[−0.0124, 0.0368]	[−0.0116, 0.0376]	[−0.0193, 0.1112]	[0.0094, 0.2019]	[0.0270, 0.2409]
High school and above	0.0311^**^	0.0310^**^	0.0335^**^	0.1262^***^	0.1957^***^	0.1688^**^
	[0.0011, 0.0612]	[0.0009, 0.0611]	[0.0035, 0.0635]	[0.0455, 0.2069]	[0.0794, 0.3120]	[0.0374, 0.3002]
Residential region	0.0644^***^	0.0627^***^	0.0588^***^	0.0282	0.0175	−0.0175
	[0.0457, 0.0831]	[0.0440, 0.0814]	[0.0401, 0.0775]	[−0.0219, 0.0782]	[−0.0570, 0.0919]	[−0.1016, 0.0665]
Economic status	0.0203^***^	0.0186^**^	0.0188^**^	0.0982^***^	0.1422^***^	0.1067^***^
	[0.0052, 0.0354]	[0.0035, 0.0337]	[0.0036, 0.0339]	[0.0542, 0.1422]	[0.0844, 0.1999]	[0.0382, 0.1752]
Work	0.0316^***^	0.0310^***^	0.0304^***^	0.0027	0.0762	0.0032
	[0.0115, 0.0516]	[0.0108, 0.0512]	[0.0102, 0.0505]	[−0.0528, 0.0581]	[−0.0189, 0.1712]	[−0.1060, 0.1124]
Living arrangement	0.0740^***^	0.0740^***^	0.0741^***^	0.0574	0.0924	0.0649
	[0.0410, 0.1070]	[0.0409, 0.1071]	[0.0410, 0.1072]	[−0.0296, 0.1445]	[−0.0441, 0.2289]	[−0.0893, 0.2191]
Chronic disease	0.0196^*^	0.0145	0.0150	0.0790^***^	0.1114^***^	0.0040
	[−0.0005, 0.0397]	[−0.0054, 0.0344]	[−0.0050, 0.0349]	[0.0206, 0.1373]	[0.0300, 0.1928]	[−0.0920, 0.1001]
Self-rated health	0.1882^***^	0.1899^***^	0.1900^***^	0.4345^***^	0.3889^***^	0.3263^***^
	[0.1788, 0.1976]	[0.1805, 0.1993]	[0.1806, 0.1994]	[0.4042, 0.4647]	[0.3445, 0.4334]	[0.2744, 0.3781]
Exercise	0.0152^*^	0.0146^*^	0.0127	−0.0015	0.0424	−0.0124
	[−0.0021, 0.0326]	[−0.0028, 0.0320]	[−0.0047, 0.0301]	[−0.0476, 0.0446]	[−0.0221, 0.1069]	[−0.0859, 0.0611]
Underweight	−0.0530^***^	−0.0515^**^	−0.0497^**^	−0.0217	−0.2180^**^	−0.1409
	[−0.0924, -0.0137]	[−0.0911, -0.0118]	[−0.0894, -0.0100]	[−0.1268, 0.0834]	[−0.3932, -0.0428]	[−0.3401, 0.0582]
Overweight	0.0542^***^	0.0533^***^	0.0528^***^	0.0466^*^	0.0880^***^	0.0235
	[0.0359, 0.0725]	[0.0350, 0.0715]	[0.0346, 0.0711]	[−0.0029, 0.0961]	[0.0214, 0.1546]	[−0.0530, 0.1001]
Obese	0.0101	0.0104	0.0078	0.0702	0.1147	0.1428
	[−0.0343, 0.0546]	[−0.0339, 0.0548]	[−0.0366, 0.0522]	[−0.0589, 0.1993]	[−0.0598, 0.2892]	[−0.0684, 0.3539]
_cons	−0.1538^***^	−0.1419^***^	−0.1403^***^	1.9490^***^	1.9563^***^	2.3010^***^
	[−0.2018, −0.1057]	[−0.1899, −0.0938]	[−0.1884, −0.0923]	[1.8029, 2.0951]	[1.7404, 2.1722]	[2.0422, 2.5597]
*N*	11,292	11,292	11,292	5,512	2,886	2,124
*R* ^2^	0.1550	0.1530	0.1535	0.2193	0.2023	0.1498
Adj. *R*^2^	0.1538	0.1517	0.1522	0.2168	0.1975	0.1429

The asterisks indicate statistical significance levels: *p < 0.1, **p < 0.05, and ***p < 0.01.

#### 3.3.2 Robustness test for replacing sample

To address potential sample selection concerns, we conducted an additional robustness check by restricting our analysis to a subsample of respondents who explicitly expressed interest in or need for community-based health services. The rationale for this approach stems from the possibility that older population who are indifferent to the availability of such services may possess inherently higher baseline life satisfaction levels. If these individuals maintain elevated satisfaction regardless of service exposure, their inclusion in the full sample could lead to an overestimation of the true treatment effects. To mitigate this concern, we identified participants based on their survey responses indicating a preference for or requirement of community health services, and re-estimated our models using only this targeted subsample. This analytical strategy provides a more conservative test of our hypotheses by focusing on individuals who are most likely to benefit from these interventions. The results from this restricted sample analysis continue to show statistically significant positive relationships between community-based health services and life satisfaction outcomes. The persistence of these effects in the subsample analysis provides additional evidence supporting the validity and robustness of our main findings.

#### 3.3.3 Robustness test for sample selectivity bias

The four matching methods were used in the present study, and the average treatment effect on the treated (ATT) difference was calculated. A balanced test of the samples revealed that the matched samples were well-balanced. After matching the treated and control groups, no significant differences were found between the two groups with respect to the variables under investigation. Furthermore, it was found that the standard deviation of all variables after the matching process was less than 10%. These results indicate that the selectivity bias of the sample is eliminated to a large extent through propensity score matching.

We designate kernel matching as our primary estimation method due to its superior performance in covariate balance quality, estimation efficiency, and sample utilization (34). Our main findings demonstrate that community-based health services significantly improve life satisfaction among older adults, with kernel matching estimates of 0.1047 for health check-ups, 0.1268 for health records, and 0.1189 for health education. The consistency of results across all four matching methods provides compelling evidence for the robustness of these findings and validates our choice of kernel matching as the primary method. Specifically, the effect estimates across methods show convergent validity, with health check-up effects ranging from 0.0924 to 0.1136, health records effects ranging from 0.0941 to 0.1502, and health education effects ranging from 0.1189 to 0.1528. These robust findings further confirm the positive impact of community-based health services on life satisfaction among older adults (Table 4).

**Table 4:** PSM results of community-based healthcare services on the life satisfaction of older adults.

Community-based health services	Matching methods^a^	Life satisfaction among older adults			
		Treated	Controls	ATT	S.E.
Health check-up (N_T_ = 3,380; NC=7912^b^)	k-nearest neighbor matching (*k* = 4)	3.7695	3.6559	0.1136^***^	0.0213
	Radius matching (cal = 0.01)	3.7694	3.6622	0.1072^***^	0.0189
	Kernel matching	3.7695	3.6648	0.1047^***^	0.0188
	Mahal matching	3.7695	3.6771	0.0924^***^	0.0208
Health records (N_T_ = 1,225; NC = 10,067)	k-nearest neighbor matching (*k* = 4)	3.7541	3.6301	0.1240^***^	0.0324
	Radius matching (cal = 0.01)	3.7539	3.6037	0.1502^***^	0.0299
	Kernel matching	3.7541	3.6273	0.1268^***^	0.0297
	Mahal matching	3.7543	3.6602	0.0941^***^	0.0301
Health education (N_T_ = 794; NC = 10,498)	k-nearest neighbor matching (*k* = 4)	3.7594	3.6158	0.1436^***^	0.0359
	Radius matching (cal = 0.01)	3.7629	3.6101	0.1528^***^	0.0327
	Kernel matching	3.7594	3.6405	0.1189^***^	0.0325
	Mahal matching	3.7594	3.6275	0.1319^***^	0.0354

^a^Matching methods include k-nearest neighbor matching, radius matching, kernel matching, and Mahalanobis matching. ^b^NT denotes the sample size of the treated group; NC denotes the sample size of the control group. The asterisks indicate statistical significance: ***p < 0.01.

### 3.4 Heterogeneity analysis

The heterogeneity analysis, presented in Table 5, reveals that the positive effects of community-based health services vary significantly across key subpopulations. A notable gender disparity emerged, with the benefits being generally more pronounced for female older adults. For health records, the ATT for females (ranging from 0.1085 to 0.1945) was substantially larger than for males (0.0734–0.1023). Furthermore, the services yielded consistently greater improvements in life satisfaction for rural residents compared to their urban counterparts, suggesting the services may be filling a more significant need in non-urban settings. The findings for chronic disease status were more nuanced: while health education was more impactful for those without chronic conditions, the establishment of health records was significantly more beneficial for older adults with chronic diseases. This indicates that the effectiveness of specific services is highly dependent on the target group’s pre-existing health profile.

**Table 5:** Heterogeneity: PSM results of community-based healthcare services on the life satisfaction of older adults.

Community-based health services	Matching methods	Life satisfaction among older adults							
		Treated	Controls	ATT	S.E.	Treated	Controls	ATT	S.E.
Gender heterogeneity		Female older adults (*N* = 5,594)				Male older adults (*N* = 5,698)			
Health check-up	k-nearest neighbor matching (*k* = 4)	3.7672	3.6726	0.0946^***^	0.0304	3.7724	3.6449	0.1275^***^	0.0300
	Radius matching (cal = 0.01)	3.7672	3.6473	0.1199^***^	0.0267	3.7724	3.6735	0.0989^***^	0.0270
	Kernel matching	3.7672	3.6481	0.1191^***^	0.0267	3.7724	3.6798	0.0926^***^	0.0267
	Mahal matching	3.7668	3.6626	0.1042^***^	0.0290	3.7724	3.6557	0.1167^***^	0.0309
Health records	k-nearest neighbor matching (*k* = 4)	3.7809	3.6155	0.1653^***^	0.0467	3.7277	3.6327	0.0950^**^	0.0450
	Radius matching (cal = 0.01)	3.7809	3.5777	0.2032^***^	0.0431	3.7273	3.6231	0.1042^**^	0.0418
	kernel matching	3.7809	3.6029	0.1780^***^	0.0427	3.7277	3.6480	0.0797^*^	0.0415
	Mahal matching	3.7813	3.6427	0.1386^***^	0.0430	3.7277	3.6682	0.0595	0.0436
Heath education	k-nearest neighbor matching (*k* = 4)	3.7564	3.5833	0.1731^***^	0.0498	3.7629	3.6489	0.1140^**^	0.0523
	Radius matching (cal = 0.01)	3.7564	3.5378	0.2186^***^	0.0453	3.7629	3.6677	0.0952^**^	0.0481
	Kernel matching	3.7564	3.5827	0.1737^***^	0.0448	3.7629	3.6954	0.0675	0.0475
	Mahal matching	3.7564	3.6259	0.1305^***^	0.0489	3.7629	3.6519	0.1110^**^	0.0528

Rural–urban heterogeneity		Rural older adults (*N* = 5,553)				Urban older adults (*N* = 5,739)			
Health check-up	k-nearest neighbor matching (*k* = 4)	3.8022	3.6601	0.1421^***^	0.0296	3.7332	3.6587	0.0745^**^	0.0304
	Radius matching (cal = 0.01)	3.8022	3.6247	0.1775^***^	0.0264	3.7353	3.6832	0.0521^*^	0.0275
	Kernel matching	3.8022	3.6131	0.1891^***^	0.0262	3.7332	3.7009	0.0322	0.0274
	Mahal matching	3.8035	3.6338	0.1697^***^	0.0296	3.7328	3.6963	0.0365	0.0306
Health records	k-nearest neighbor matching (*k* = 4)	3.7836	3.6371	0.1465^***^	0.0448	3.7292	3.6321	0.0971^**^	0.0468
	Radius matching (cal = 0.01)	3.7836	3.5912	0.1924^***^	0.0412	3.7292	3.6199	0.1093^**^	0.0438
	Kernel matching	3.7836	3.5990	0.1846^***^	0.0407	3.7292	3.6530	0.0762^*^	0.0435
	Mahal matching	3.7840	3.6498	0.1342^***^	0.0434	3.7248	3.6954	0.0293	0.0441
Heath education	k-nearest neighbor matching (*k* = 4)	3.8526	3.6671	0.1855^***^	0.0633	3.7159	3.5779	0.1380^***^	0.0437
	Radius matching (cal = 0.01)	3.8526	3.6490	0.2036^***^	0.0582	3.7159	3.5895	0.1264^***^	0.0399
	Kernel matching	3.8526	3.6528	0.1998^***^	0.0574	3.7159	3.6321	0.0838^**^	0.0396
	Mahal matching	3.8532	3.6627	0.1905^***^	0.0596	3.7159	3.6310	0.0849^*^	0.0446

Chronic disease heterogeneity		Older adults without chronic disease (*N* = 8,876)				Older adults with chronic disease (*N* = 2,416)			
Health check-up	k-nearest neighbor matching (*k* = 4)	3.7229	3.6278	0.0951^***^	0.0231	4.0397	3.9232	0.1165^**^	0.0500
	Radius matching (cal = 0.01)	3.7232	3.6172	0.1060^***^	0.0207	4.0397	3.9133	0.1264^***^	0.0448
	Kernel matching	3.7229	3.6189	0.1040^***^	0.0206	4.0397	3.9220	0.1177^***^	0.0444
	Mahal matching	3.7231	3.6316	0.0914^***^	0.0224	4.0475	3.9153	0.1322^***^	0.0486
Health records	k-nearest neighbor matching (*k* = 4)	3.7059	3.5938	0.1121^***^	0.0353	4.0745	3.8966	0.1779^**^	0.0739
	Radius matching (cal = 0.01)	3.7059	3.5600	0.1459^***^	0.0328	4.0745	3.8895	0.1850^***^	0.0665
	Kernel matching	3.7059	3.5772	0.1287^***^	0.0326	4.0745	3.9125	0.1620^**^	0.0652
	Mahal matching	3.7074	3.6171	0.0903^***^	0.0329	4.0617	3.8935	0.1682^**^	0.0665
Heath education	k-nearest neighbor matching (k = 4)	3.7363	3.5978	0.1385^***^	0.0382	3.9875	3.9075	0.0800	0.1005
	Radius matching (cal = 0.01)	3.7363	3.5743	0.1620^***^	0.0349	3.9875	3.9163	0.0712	0.0920
	Kernel matching	3.7363	3.5997	0.1366^***^	0.0346	3.9875	3.9268	0.0606	0.0902
	Mahal matching	3.7363	3.6059	0.1304^***^	0.0393	3.9630	3.8704	0.0926	0.0997

Female subsample: For health check-up (N_T_ = 1741; NC = 3,853); health records (N_T_ = 608; NC = 4,986); and health education (N_T_ = 427; NC = 5,167). Male subsample: For health check-up (N_T_ = 1,639; NC = 4,059); health records (N_T_ = 617; NC = 5,081); and health education (N_T_ = 367; NC = 5,331). Rural subsample: For health check-up (N_T_ = 1756; NC = 3,797); health records (N_T_ = 611; NC = 4,942); and health education (N_T_ = 252; NC = 5,301). Urban subsample: For health check-up (N_T_ = 1,624; NC = 4,115); health records (N_T_ = 614; NC = 5,125); and health education (N_T_ = 542; NC = 5,197). Subsample without chronic disease: For health check-up (N_T_ = 2,896; NC = 5,980); health records (N_T_ = 1,063; NC = 7,813); and health education (N_T_ = 713; NC = 8,163). Subsample with chronic disease: For health check-up (N_T_ = 484; NC = 1932); health records (N_T_ = 162; NC = 2,254); and health education (N_T_ = 81; NC = 2,335). The asterisks indicate statistical significance levels: *p < 0.1, **p < 0.05, and ***p < 0.01.

## 4 Discussion

Community-based health services have a significant wellbeing promoting effect through multiple pathways. In China, these services primarily provide preventive and supportive interventions for community-dwelling older adults, focusing mainly on health maintenance and early detection of health problems. Therefore, community-based health services play a positive role in promoting healthcare utilization and facilitating social participation (35).

Firstly, community-based health services effectively reduce barriers to seeking medical care, mitigate treatment delays, and thus address unmet health needs, increasing healthcare utilization and improving health outcomes. Community health providers act as a “bridge” between healthcare institutions and home-dwelling older adults, facilitating communication with healthcare professionals, offering health consultations, and encouraging regular health monitoring, significantly reducing barriers to accessing healthcare services (36, 37). Additionally, older adults participating in community-based health services for preventive purposes are more likely to be referred to medical facilities and follow prescribed treatment plans. In this way, community health providers also serve as “health managers” for community-dwelling older adults.

Secondly, community-based health services create opportunities to promote social interaction and enhance social integration. Through these services, older adults engage in regular interactions with healthcare providers, peers, and community members (38). These interactions and educational activities provide effective psychological comfort and social support, helping to maintain and expand social networks. The social support generated through these interactions fosters a shared sense of community identity and collective values in a familiar living environment, making socially isolated older adults feel more connected and empowered to engage in social activities. Participation in community health programs and interpersonal communication are important channels for older adults to seek emotional connections and maintain mental wellbeing; active social participation helps maintain positive emotions and life satisfaction.

The heterogeneity analysis reveals important differential impacts across demographic subgroups. Regarding gender, community-based health services provide greater life satisfaction improvements for female older adults than for male older adults. In traditional “male breadwinner, female homemaker” labor divisions and considering historical educational inequalities, males typically enjoy higher social status and more economic resources, which they can leverage in securing care arrangements. This gives them greater autonomy in accessing various health services, potentially reducing their relative reliance on community-based programs. In contrast, females have historically invested more time and energy into family care responsibilities but may have fewer economic resources and social connections to access diverse health services. As a result, female older adults are more responsive to community-based health services and derive greater satisfaction benefits from these accessible, community-centered interventions.

The pronounced effectiveness of community health services among rural older adults, particularly for health education programs, reflects several contextual mechanisms specific to rural environments. Rural areas typically experience significant healthcare resource constraints, limited access to specialized health information, and reduced availability of formal health promotion programs. In this context, community-based health education fills critical knowledge gaps and provides essential health management skills that are otherwise unavailable. The community-centered delivery approach aligns particularly well with rural social structures, where tight-knit community networks facilitate information sharing, peer support, and collective health behavior change. Rural older adults may also have stronger community identity and social cohesion that amplifies the social benefits derived from group-based health education activities, creating synergistic effects between health knowledge acquisition and social participation enhancement.

The chronic disease heterogeneity analysis reveals a striking and policy-significant pattern that demonstrates the importance of targeted service delivery approaches. For older adults with chronic conditions, health records establishment emerges as the most beneficial intervention, with ATT ranging up to 0.1850 across different matching methods. This substantial effectiveness reflects the critical importance of systematic health information management, care coordination, and longitudinal monitoring for individuals managing complex, ongoing health conditions. Health records enable healthcare providers to track disease progression, medication adherence, and treatment responses over time, facilitating evidence-based care adjustments and preventing dangerous gaps in care continuity. In sharp contrast, older adults without chronic diseases derive significantly greater benefits from health education programs, with ATT values reaching up to 0.1620. This pattern indicates that for healthy aging populations, knowledge-based interventions focused on prevention, lifestyle modification, and health maintenance represent the most effective pathway to enhanced life satisfaction. Health education empowers these individuals with the tools and information necessary to maintain their current health status, adopt protective behaviors, and prevent the onset of chronic conditions. This differential effectiveness pattern has profound implications for community health service design and resource allocation. The findings strongly support a stratified approach to service delivery, where preventive services such as health education should be prioritized and expanded for healthy aging populations, while management-oriented services, particularly comprehensive health records systems, must be emphasized for those with chronic conditions. This evidence-based differentiation ensures that limited community health resources are deployed most effectively, maximizing population health outcomes by matching service types to population health profiles. Such targeted approaches represent a fundamental shift from one-size-fits-all community programming toward precision public health interventions that recognize the heterogeneous needs of aging populations.

The differential effectiveness patterns observed across the three community health services warrant detailed mechanistic explanation, particularly the paradoxical finding that health education demonstrates the highest treatment effects despite having the lowest utilization rates. This apparent contradiction can be understood through several interconnected pathways related to service content, delivery characteristics, and participant selection dynamics. Health education programs achieve superior effectiveness through their comprehensive, multi-dimensional approach to health promotion. This contrasts sharply with the other services. For example, while health check-ups focus on clinical assessment, existing literature suggests these free services often suffer from homogeneity, focusing on basic screening without targeted, individualized risk assessment or strong follow-up management (i.e., “check-ups without management”). In contrast, health education is a deep intervention that addresses the root causes of wellbeing: it is not just a one-time screening but a behavioral and cognitive intervention that enhances health literacy, builds self-efficacy, and fosters social connections. The interactive and personalized nature of these programs creates intensive exposure effects that generate substantial improvements in participants’ knowledge, attitudes, and behaviors. Furthermore, health education sessions typically involve group-based learning formats that foster peer support networks and social connections, addressing both health knowledge gaps and social isolation concerns that significantly impact life satisfaction among older adults. The low utilization rate of health education programs, while concerning from a population health perspective, may contribute to their high measured effectiveness through positive selection mechanisms. The voluntary and proactive nature of health education participation tends to attract older adults with higher health motivation, better baseline health awareness, and stronger commitment to behavioral change. These individuals are inherently more likely to derive substantial benefits from educational interventions and implement recommended lifestyle modifications. Additionally, the accessibility barriers associated with health education programs, including scheduling requirements, transportation needs, and sustained participation demands, may result in a self-selected population of highly engaged participants who maximize the potential benefits of these interventions.

Our findings provide strong empirical support for both Healthy Aging and Active Aging theoretical frameworks. The positive effects of all three community-based health services demonstrate how these interventions contribute to the core pillars of healthy aging through health optimization, social participation facilitation, and security enhancement. The heterogeneity analysis particularly illuminates how active aging principles operate differently across population subgroups, with health education supporting preventive care for healthier older adults and health records enabling continued autonomy for those managing chronic conditions. These differential effects underscore the importance of tailored approaches to implementing healthy and active aging frameworks in practice.

The findings from this study have significant implications beyond the Chinese context, offering valuable insights for other countries confronting similar demographic challenges. As populations age globally, many nations are exploring community-based approaches to support aging in place and reduce reliance on institutional care. Our results provide empirical evidence supporting the effectiveness of comprehensive community health services in enhancing older adult wellbeing, particularly for vulnerable populations who may have limited access to formal healthcare services. The differential effects observed across demographic subgroups highlight the importance of considering population heterogeneity in designing and implementing care for older adults. Countries with diverse populations or significant rural–urban disparities may benefit from adopting targeted approaches similar to those recommended for China.

Based on our empirical findings, several evidence-based policy recommendations emerge to enhance the effectiveness of community-based health services for older adults in China. First, establishing comprehensive service quality assurance and monitoring systems represents a fundamental priority. While our study demonstrates significant associations between service utilization and life satisfaction, the binary measurement approach highlights the critical need for robust quality standards and outcome metrics. Future policy expansion should prioritize implementing standardized service quality evaluation frameworks, developing evidence-based care protocols, and establishing real-time monitoring systems to ensure that older adults can effectively access these services and have their needs met. These quality assurance mechanisms should include standardized outcome indicators, regular service effectiveness assessments, and continuous quality improvement processes. Second, our heterogeneity analysis revealing differential effectiveness patterns across chronic disease status provides compelling evidence for differentiated service delivery strategies. For older adults with chronic conditions, health record establishment demonstrates substantially greater benefits, with Average Treatment Effects on the Treated ranging up to 0.1850, reflecting the critical importance of systematic health information management and longitudinal care coordination. Conversely, older adults without chronic diseases derive significantly greater benefits from health education programs, with ATT values reaching up to 0.1620, emphasizing the effectiveness of preventive interventions for healthy aging populations. This evidence supports implementing targeted service allocation policies that prioritize management-oriented services for chronically ill populations while emphasizing prevention-oriented services for healthy older adults. Third, addressing the substantial disparities in service utilization rates represents an urgent policy imperative. The particularly low utilization of health education services at only 7.03%, despite their demonstrated effectiveness, reveals significant gaps in service accessibility and delivery mechanisms. Policy interventions should focus on expanding service coverage, particularly for health education programs, developing innovative service delivery models that enhance convenience and accessibility, and implementing proactive outreach strategies to reach underserved populations. Digital health platforms and community-based service networks can play crucial roles in improving service accessibility and reducing barriers to utilization. Fourth, the post-pandemic context has further heightened the importance of community-based health services as essential components of resilient healthcare systems. Policy frameworks should strengthen community health service capacity for emergency response, develop hybrid online-offline service delivery models to ensure service continuity during health emergencies, and enhance the role of community-based services in maintaining healthcare access when traditional healthcare systems face disruptions. These adaptations are particularly crucial for older adults who may face greater barriers to accessing conventional healthcare services during crisis periods. Finally, successful implementation of these recommendations requires establishing robust inter-sectoral coordination mechanisms involving health, civil affairs, and social insurance departments to ensure unified policy development and implementation. This comprehensive approach, grounded in our empirical findings, will enable more effective resource allocation, improved service targeting, and enhanced outcomes for China’s rapidly aging population.

Several limitations of this study should be acknowledged when interpreting the findings. First, the cross-sectional nature of the CLASS dataset limits our ability to establish definitive causal relationships and may not fully capture the long-term dynamic effects of community-based health services on life satisfaction. While our PSM procedures help balance observable characteristics between treatment and control groups, unmeasured confounders may still influence the results. Therefore, our findings should be interpreted as strong associations consistent with beneficial effects rather than efinitive causal relationships. Second, our measurement of community-based health services represents a crucial limitation that requires careful interpretation. Our binary operationalization (utilization vs. non-utilization) does not capture essential service dimensions including quality variations, intervention intensity, frequency of contact, duration of engagement, or specific content delivered. This measurement restriction has several important implications for interpreting our findings. The ATT values reported in Table 5, for instance, represent average associated benefits across heterogeneous service delivery models that may vary substantially in their actual implementation and effectiveness. Consequently, our estimates may mask significant variation in outcomes between high-quality and low-quality service provision. The observed associations may be driven by the most effective implementations, potentially overestimating average program associations; conversely, poor-quality implementations may dilute overall association estimates, potentially underestimating the benefits of well-delivered services. Third, the community-based health services discussed in this study refer primarily to health-focused interventions and do not include broader social support services such as emotional counseling or recreational activities. Therefore, our results may underestimate the full benefits of comprehensive community-based older adult care programs. Finally, the assessments of life satisfaction were based on self-reports from older adults, which could be influenced by their feelings and circumstances at the time of the interview. Recall bias and social desirability bias may also affect the accuracy of the study results to some extent.

## 5 Conclusion

This study provides robust empirical evidence of significant positive associations between community-based health services, including health check-ups, health records management, and health education programs, and life satisfaction among older adults in China. Using data from the China Longitudinal Aging Social Survey and employing OLS regression and propensity score matching methods, our findings reveal strong correlations that are consistent with beneficial program effects, demonstrating particularly pronounced associations for female older adults, rural residents, and those without chronic diseases. These relationships hold up even after selection bias has been rigorously statistically mitigated through comprehensive propensity score matching procedures. The findings indicate that community health services are associated with multiple pathways that may contribute to enhanced wellbeing, including improved physical health conditions, enhanced social participation, and strengthened community belonging. These robust associations support the importance of expanding community-based health service coverage and developing targeted implementation strategies that consider demographic heterogeneity to optimize outcomes for China’s rapidly aging population.

## Data Availability

The original contributions presented in the study are included in the article/supplementary material, further inquiries can be directed to the corresponding author.

## References

[1] Yu Y Zhang J Petrovic M Zhang X Zhang W-H. Utilization of home- and community-based services among older adults worldwide: a systematic review and meta-analysis. Int J Nurs Stud. (2024) 155:104774. doi: 10.1016/j.ijnurstu.2024.104774, 38703696

[2] Gruber J Lin M Yang H Yi J. China’s social health Insurance in the era of rapid population aging. JAMA Health Forum. (2025) 6:e251105. doi: 10.1001/jamahealthforum.2025.1105, 40279110

[3] Tang L Yang J Zheng J Sun X Cheng L He K . Relaxing fertility policies and delaying retirement age increase China’s carbon emissions. Nat Clim Chang. (2024) 14:1228–9. doi: 10.1038/s41558-024-02145-5

[4] Wu H Wang Y Zhang H Yin X Wang L Wang L . An investigation into the health status of the elderly population in China and the obstacles to achieving healthy aging. Sci Rep. (2024) 14:31123. doi: 10.1038/s41598-024-82443-2, 39730900 PMC11681201

[5] Long C Huang J Liu D Liu C Wu M Wu H . Prevalence, combination patterns, and quality of life factors of multimorbidity among older adults in southern China based on the health ecological model. J Glob Health. (2025) 15:04215. doi: 10.7189/jogh.15.04215, 40708322 PMC12290435

[6] Ju C Liu H Gong Y Guo M Ge Y Liu Y . Changes in patterns of multimorbidity and associated with medical costs among Chinese middle-aged and older adults from 2013 to 2023: an analysis of repeated cross-sectional surveys in Xiangyang, China. Front Public Health. (2024) 12:1403196. doi: 10.3389/fpubh.2024.1403196, 39171301 PMC11335498

[7] Gardner WM Razo C McHugh TA Hagins H Vilchis-Tella VM Hennessy C . Prevalence, years lived with disability, and trends in anaemia burden by severity and cause, 1990–2021: findings from the global burden of disease study 2021. Lancet Haematol. (2023) 10:e713–34. doi: 10.1016/S2352-3026(23)00160-6, 37536353 PMC10465717

[8] Gu S Jia C Shen F Wang X Wang X Gu H. The effects of community-based home health care on the physical and mental health of older adults with chronic diseases. Qual Life Res. (2024) 33:691–703. doi: 10.1007/s11136-023-03555-2, 38032396

[9] Xu Y Hu T Wang J. How do older parents with one child live? The well-being of Chinese elders affected by the one-child policy. SAGE Open. (2024) 14:21582440241253406. doi: 10.1177/21582440241253406

[10] Jin F Chen T. Community nursing on subjective well-being of the elderly: evidence from CLASS data. BMC Nurs. (2025) 24:757. doi: 10.1186/s12912-025-03205-7, 40597969 PMC12211858

[11] Cai Y Feng W. The social and sociological consequences of China’s one-child policy. Annu Rev Sociol. (2021) 47:587–606. doi: 10.1146/annurev-soc-090220-032839

[12] Wang R Yan Z Liang Y Tan ECK Cai C Jiang H . Prevalence and patterns of chronic disease pairs and multimorbidity among older Chinese adults living in a rural area. PLoS One. (2015) 10:e0138521. doi: 10.1371/journal.pone.0138521, 26394368 PMC4578976

[13] Chen Y Ji H Shen Y Liu D. Chronic disease and multimorbidity in the Chinese older adults’ population and their impact on daily living ability: a cross-sectional study of the Chinese longitudinal healthy longevity survey (CLHLS). Arch Public Health. (2024) 82:17. doi: 10.1186/s13690-024-01243-2, 38303089 PMC10832143

[14] Chen Y Shi L Zheng X Yang J Xue Y Xiao S . Patterns and determinants of multimorbidity in older adults: study in health-ecological perspective. Int J Environ Res Public Health. (2022) 19:16756. doi: 10.3390/ijerph192416756, 36554647 PMC9779369

[15] Bao J Zhou L Liu G Tang J Lu X Cheng C . Current state of care for the elderly in China in the context of an aging population. BST. (2022) 16:107–18. doi: 10.5582/bst.2022.01068, 35431289

[16] Sen Q Lei Z. The impact of community care services on older people’s psychological health: an empirical study in Liaoning Province, China. Front Public Health. (2023) 11:1199830. doi: 10.3389/fpubh.2023.1199830, 37601200 PMC10436539

[17] Wang X q Yang C c Sun X l. Integrated physical and mental management for China’s elderly: insights from Long-Gang District, Shenzhen. Front Aging. (2024) 5:1361098. doi: 10.3389/fragi.2024.1361098, 38379538 PMC10876994

[18] Di X Wang L. The impact of accessibility of community elderly care services on quality of life of the elderly. Healthcare. (2025) 13:99. doi: 10.3390/healthcare13020099, 39857125 PMC11764981

[19] Ng ST Tey NP Asadullah MN. What matters for life satisfaction among the oldest-old? Evidence from China. PLoS One. (2017) 12:e0171799. doi: 10.1371/journal.pone.0171799, 28187153 PMC5302476

[20] Steptoe A Deaton A Stone AA. Subjective wellbeing, health, and ageing. Lancet. (2015) 385:640–8. doi: 10.1016/S0140-6736(13)61489-0, 25468152 PMC4339610

[21] Zhang J Zhang Y Wu Z Fu X. Enhancing understanding of healthy aging based on time-varying dependencies among multidimensional health, life satisfaction, and health behaviors of older adults aged 60 years and over. BMC Public Health. (2024) 24:192. doi: 10.1186/s12889-024-17752-2, 38229050 PMC10790531

[22] Zhang K Pei J Wang S Rokpelnis K Yu X. Life satisfaction in China, 2010–2018: trends and unique determinants. Appl Res Qual Life. (2022) 17:2311–48. doi: 10.1007/s11482-021-10031-x

[23] Zhang Y Sun L. The health status, social support, and subjective well-being of older individuals: evidence from the Chinese general social survey. Front Public Health. (2024) 12:1312841. doi: 10.3389/fpubh.2024.1312841, 38333739 PMC10850324

[24] Xu Z Zhang W Zhang X Wang Y Chen Q Gao B . Multi-level social capital and subjective wellbeing among the elderly: understanding the effect of family, workplace, community, and society social capital. Front Public Health. (2022) 10:772601. doi: 10.3389/fpubh.2022.772601, 35493385 PMC9051067

[25] Thiam Y Allaire J-F Morin P Hyppolite S-R Doré C Zomahoun HTV . A conceptual framework for integrated community care. Int J Integr Care. 21:5. doi: 10.5334/ijic.5555, 33613137 PMC7880006

[26] Ronzi S Orton L Pope D Valtorta NK Bruce NG. What is the impact on health and wellbeing of interventions that foster respect and social inclusion in community-residing older adults? A systematic review of quantitative and qualitative studies. Syst Rev. (2018) 7:26. doi: 10.1186/s13643-018-0680-2, 29382375 PMC5789687

[27] Rudnicka E Napierała P Podfigurna A Męczekalski B Smolarczyk R Grymowicz M. The World Health Organization (WHO) approach to healthy ageing. Maturitas. (2020) 139:6–11. doi: 10.1016/j.maturitas.2020.05.018, 32747042 PMC7250103

[28] Boudiny K. Active ageing’: from empty rhetoric to effective policy tool. Ageing Soc. (2013) 33:1077–98. doi: 10.1017/S0144686X1200030X, 23913994 PMC3728916

[29] Zhao D Zhou Z Shen C Zhai X Zhao Y Cao D . The effect of health check-ups on health among the elderly in China: evidence from 2011–2018 longitudinal data. Int J Public Health. (2022) 67:1604597. doi: 10.3389/ijph.2022.1604597, 35990189 PMC9389946

[30] Brownson RC Haire-Joshu D Luke DA. SHAPING THE CONTEXT OF HEALTH: a review of environmental and policy approaches in THE prevention of chronic diseases. Annu Rev Public Health. (2006) 27:341–70. doi: 10.1146/annurev.publhealth.27.021405.102137, 16533121

[31] Artinian NT Fletcher GF Mozaffarian D Kris-Etherton P Van Horn L Lichtenstein AH . Interventions to promote physical activity and dietary lifestyle changes for cardiovascular risk factor reduction in adults: a scientific statement from the American Heart Association. Circulation. (2010) 122:406–41. doi: 10.1161/CIR.0b013e3181e8edf1, 20625115 PMC6893884

[32] Cheung HY Fu YQ Xu SC Yang Z. Older people’s decisions of the community care: a welfare pluralism perspective. Humanit Soc Sci Commun. (2025) 12:435. doi: 10.1057/s41599-025-04487-7

[33] Zeng Y Poston DL Vlosky DA Gu D. Healthy longevity in China: demographic, socioeconomic, and psychological dimensions. Dordrecht: Springer (2008).

[34] Caliendo M Kopeinig S. Some practical guidance for the implementation of propensity score matching. J Econ Surv. (2008) 22:31–72. doi: 10.1111/j.1467-6419.2007.00527.x

[35] Gu J Wang Q Qiu W Wu C Qiu X. Chronic diseases and determinants of community health services utilization among adult residents in southern China: a community-based cross-sectional study. BMC Public Health. (2024) 24:919. doi: 10.1186/s12889-024-18435-8, 38549080 PMC10979594

[36] Fisher EM Akiya K Wells A Li Y Peck C Pagán JA. Aligning social and health care services: the case of community care connections. Prev Med. (2021) 143:106350. doi: 10.1016/j.ypmed.2020.106350, 33253760

[37] Kennedy MA Hatchell KE DiMilia PR Kelly SM Blunt HB Bagley PJ . Community health worker interventions for older adults with complex health needs: a systematic review. J Am Geriatr Soc. (2021) 69:1670–82. doi: 10.1111/jgs.17078, 33738803 PMC8263299

[38] Chua CMS Chua JYX Shorey S. Effectiveness of home-based interventions in improving loneliness and social connectedness among older adults: a systematic review and meta-analysis. Aging Ment Health. (2024) 28:1–10. doi: 10.1080/13607863.2023.2237919, 37466183

[39] Ferrer‐i‐Carbonell A Frijters P. How Important is Methodology for the Estimates of the Determinants of Happiness? The Economic Journal. (2004) 114:641–659. doi: 10.1111/j.1468-0297.2004.00235.x, 38333739

[40] World Health Organization. Men, ageing and health : achieving health across the life span. World Health Organization. World Health Organization. (2001). Available at: https://iris.who.int/handle/10665/66941

